# Optimizing Physical Fitness Before Colorectal Cancer Surgery (CANOPTIPHYS): The Effect of Preoperative Exercise on Pre- and Postoperative Physical Fitness in Older people – A Randomized Controlled Trial

**DOI:** 10.1177/21501319251346417

**Published:** 2025-06-17

**Authors:** Johanna Danielsson, Julia Engström Sid, Mikael Andersson, Malin Nygren-Bonnier, Monika Egenvall, Maria Hagströmer, Laura E. Vossen, Ing-Mari Dohrn, Elisabeth Rydwik

**Affiliations:** 1 Karolinska Institutet, Huddinge, Sweden; 2 Uppsala University, Sweden; 3 Karolinska University Hospital, Stockholm, Sweden; 4 Karolinska Institutet, Stockholm, Sweden; 5 Academic Primary Health Care Centre, Region Stockholm, Sweden; 6 Sophiahemmet University, Stockholm, Sweden

**Keywords:** preoperative exercise, physical function, prehabilitation, cancer surgery, colorectal surgery, postoperative recovery

## Abstract

**Introduction/Objective:**

Surgery-induced losses in physical fitness may have detrimental consequences for patients with low reserve capacity at start. Our objective was to evaluate the effect of preoperative exercise on physical fitness in older patients with low physical fitness scheduled for colorectal cancer surgery.

**Methods:**

In this randomized, controlled trial, patients ≥65 years of age, who were scheduled for colorectal cancer surgery were included if they had a low maximal walking speed. Exercise for 2 to 3 weeks before surgery was home-based, high-intensity, and partly supervised by a primary care physiotherapist. The intervention consisted of inspiratory muscle training, aerobic, and strength exercises. A control group underwent usual preoperative care. Physical fitness was assessed with the 6-min walk test (primary outcome), the 30-s chair stand test and maximal inspiratory pressure which estimates inspiratory muscle strength (secondary outcomes). The effect of preoperative exercise on these outcomes before and after surgery was analyzed with linear mixed-models for the 6-min walk test and maximal inspiratory pressure and with a non-parametric Friedman rank sum test for the 30-s chair stand test. To evaluate adherence, physical activity was measured and compared for both groups.

**Results:**

A total of 52 participants were included. Analyses showed a statistically significant effect of preoperative exercise on postoperative inspiratory muscle strength. We did not find an effect of preoperative exercise on 6-min walk test or 30-s chair stand test performance. Regarding preoperative physical activity, the intervention group engaged more in high-intensity physical activity in relation to their total stepping time compared to the control group.

**Conclusions:**

Short-term exercise before colorectal cancer surgery can provide benefits in terms of increased postoperative inspiratory muscle strength in older patients with low physical fitness. While we could not demonstrate an effect of preoperative exercise on any other outcomes, these results should be interpreted with caution due to a small sample size.

**Trial registration:**

Clinicaltrials.gov, identification number: NCT04878185, URL: Study Details | Optimizing Physical Function Before Cancer Surgery in Older People at Risk | ClinicalTrials.gov.

## Introduction

Among patients undergoing colorectal cancer surgery in Sweden, 62% are ≥70 years of age.^1^ Older age may be accompanied by reduced reserve capacity to tolerate the surgical trauma.^2,3^ Furthermore, neoadjuvant treatments and prolonged bed rest associated with major surgery, contribute to cardiorespiratory and muscular deconditioning.^4-6^ Older patients face significant reductions in functional capacity and lower extremity strength after major abdominal surgery,^7^ increasing their risk of withstanding disability.^8-10^ Nevertheless, older adults are a heterogenous population with wide-ranging levels of reserve capacity, where those with lower physical fitness before major abdominal surgery are at higher risk of dependence in ambulation after surgery.^7^ Colorectal cancer surgery is typically planned, allowing for targeted efforts that may mitigate the surgery-related losses in physical fitness.^11-13^

A systematic review and meta-analysis found no benefits of exercise-based interventions on functional capacity, measured with the 6-min walk test (6MWT), for frail patients undergoing colorectal cancer surgery, possibly due the low number of studies included or insufficient exercise intensity.^14^ In contrast, a systematic review and meta-analysis, not limited to high-risk patients, showed a significant effect of preoperative exercise on functional capacity.^15^ Nevertheless, most studies applied interventions lasting over 3 weeks,^15^ which may be difficult to implement in some countries due to short cancer treatment target intervals.^16^ Furthermore, preoperative exercise interventions often vary in content (eg, multimodal or unimodal interventions), intensity, frequency, and setting. For older patients with low physical fitness, the home-environment can facilitate program adherence by removing transportation barriers,^17^ and allow exercises tailored to overcome mobility restrictions in everyday life.^18^ However, the effect of home-based exercise before colorectal cancer surgery on postoperative physical fitness remains uncertain.^19^

A home-based, high-intensity, high-frequency preoperative exercise program was previously adapted to fit the cancer treatment target times in Sweden, stipulating a maximum of 21 days between “decision to treat” and the start of treatment. The intervention was deemed feasible regarding acceptance and adherence among participants,^20^ and we hypothesized that it would generate improvements in preoperative physical fitness, resulting in better postoperative physical fitness. Therefore, the aim of this study was to evaluate the effect of preoperative exercise compared to usual care, on changes in physical fitness throughout the perioperative period, in older patients with low physical fitness. Additionally, we aimed to provide robust information on adherence, by measuring physical activity and sedentary behavior during the preoperative phase.

## Methods

### Study Design and Setting

This was a parallel group, randomized controlled, multicenter trial where participants were recruited from 4 different hospitals in the Stockholm region: South General Hospital, Ersta Hospital, and Karolinska University Hospital in Solna and Huddinge. Pre-registration of this trial can be found at ClinicalTrials.gov, reg. no: NCT04878185 (registered April 6, 2021). Ethical approval was obtained from the Regional Ethical Review Board in Stockholm [2015/1179-31] with additional amendments [2016/1587-32, 2017/1246-32, 2021/00335, 2021-01248, 2022-00993-02]. This study is reported in accordance with Consolidated Standards of Reporting Trials (CONSORT) guidelines.^21^ The study design, procedure, and intervention have been detailed in a previously published study protocol.^22^

### Participants

Adults aged ≥65 years undergoing elective surgery due to colorectal adenocarcinoma and/or liver metastases were included if they had a maximal walking speed <2 m/s,^23^ spoke and understood the Swedish language and were residents in Stockholm County. Exclusion criteria encompassed a health status or a planned surgical procedure that inhibited participation. Furthermore, if planning surgery after >2 weeks entailed a medical risk, the patient was not considered eligible.^22^ Recruiting sites follow enhanced recovery programs and provide routine nutritional screening and treatment.

### Study Procedure

Eligible patients were screened for maximal walking speed and initially informed about the study on the first visit at the surgical out-patient clinic. An informative follow-up call was made where the patient was invited to ask questions and, if verbal consent was received, a baseline visit was scheduled. Written informed consent was collected before the start of any baseline assessments.

The outcome assessors, physiotherapists, or research nurses at each recruitment site, were blinded to group assignment and the participants were instructed not to inform the assessor about their allocation. Study-specific education was provided to the outcome assessors and primary care physiotherapists managing the intervention and included digital workshops and practical training. The assessor’s education was focused on the outcome measurements to ensure standardization, and they were provided with written instructions and a checklist. The education for the primary care physiotherapists focused on specific exercise considerations for older adults and during cancer treatment as well as lung physiology in relation to IMT. Additionally, they received education on the specific intervention, along with written instructions and a checklist. All study personnel were encouraged to keep a close dialog with the trial management group throughout the study, and follow-up meetings were held to exchange experiences and discuss challenges.^22^

Due to the low recruitment rate in the feasibility study, we addressed 2 common reasons for declined participation. Transportation issues was resolved by offering taxi service to and from preoperative assessments. Concerns about delaying surgery to make time for preoperative exercise was addressed by emphasizing patient information from the clinician that a delay of 1 to 2 weeks was not associated with a medical risk. Furthermore, we recruited from 4 sites compared to 1 site in the feasibility study. Data was stored with Research Electronic Data Capture (REDCap) hosted by Karolinska Institutet.^24,25^

### Randomization

Study enrolment was managed by on-site nurses or physiotherapists, while the randomization was performed by the trial management group. Participants were randomized into an intervention group or a control group with a permuted block randomization, using computer-generated random number tables applying a 1:1 allocation ratio. To reduce the risk of predicting group allocation, blocks were randomly ordered in different sizes. The randomization was performed after the baseline assessment and participants were informed about their allocation by telephone. For more information on the randomization process, see the study protocol.^22^

### Intervention

During the 2 to 3 weeks before surgery, participants in the intervention group were planned to receive at least 6 supervised training sessions. The home-based, high-intensity, and progressive intervention included 3 blocks targeting different areas of physical fitness: inspiratory muscle training (IMT; Block I), aerobic exercises (Block II), and functional strength exercises focused on lower extremity strength (Block III).^22^

IMT was performed with 30 breaths, starting at 50% of maximal inspiratory pressure, with a target intensity of 5 to 7 on the Borg CR-10 Scale.^26^ Aerobic exercises, such as walking or stair climbing were performed in intervals to allow for shorter bouts of higher intensity training. Functional strength training included step-up and chair rise exercises. Both aerobic and strength exercises had a target intensity of 7 to 8 on the Borg CR-10 Scale, and for these blocks, the Patient-Specific Functional Scale (PSFS) was incorporated. The PSFS is designed to quantify activity limitations, in meaningful activities self-selected by the patient,^27^ and was used to support tailored exercises for the intervention group. Progression of exercises could consist of increased resistance (Block I), increased length of intervals (Block II), and using weight belts or adding more weights (Block III). Supervised sessions were approximately 1 h long. In addition to supervised sessions, participants were instructed to include unsupervised training to reach the target frequency of IMT twice daily, and aerobic and strength training 5 to 6 times per week.

Physical activity and sedentary behavior were measured with accelerometers in both groups. As a motivational measure to increase physical activity in the intervention group, the accelerometer was omitted half-way through the intervention to provide feedback on physical activity patterns.^28^ Both groups were encouraged to achieve 150 min/week of moderate-intensity physical activity, according to physical activity guidelines for older adults.^29^ Besides the general recommendations, the control group underwent usual care. Adverse events (AEs) in the intervention group were recorded by the physiotherapists using a specific AE protocol.^22^

### Adherence

Information on type, duration, number of repetitions, and intensity of conducted exercises was documented for the supervised sessions in an exercise protocol by the physiotherapist. For the unsupervised sessions, this information was recorded in a training diary by the participant. During the preoperative period, free-living physical activity and sedentary behavior were measured for both groups with activPAL micro (PAL Technologies Ltd., Glasgow, UK). The activPAL is a thigh worn, lightweight device that measures both acceleration and body-position, and has shown good accuracy in measuring sedentary behavior, sit-to-stand transitions, and step count.^30-32^ To allow continuous 24-h monitoring, the activPAL was sealed with a shower-proof cover.

The PALanalysis software (PAL Technologies, 50 Richmond Street, Glasgow, Scotland) was used for activPAL data management and data were analyzed using PAL batch V8.11.1.63. The variables used for the analysis were selected based on the intervention: steps (n), sit-to-stand transitions (n), time standing (min), time stepping (min), time stepping 75 to 100 steps/min (min), time stepping >100 steps/min (min), time in sedentary behavior (min), and waking hours (min). Stepping 75 to 100 steps/min was considered moderate-intensity physical activity,^33^ and stepping >100 steps/min was considered high-intensity physical activity,^34^ these 2 variables were additionally analyzed as percentage of total stepping time. To account for between-group differences in waking hours, additional variables were analyzed as percentage of waking hours.

Loss of activPAL data, primarily due to technical issues, limited the analysis of the full preoperative period. To address this, 3 to 5 consecutive days with valid data were included, and the mean of these days was calculated for each variable. In total, there were 19 participants in each group (38 participants in total) with valid accelerometer data.

### Baseline Characteristics

Data regarding, age, sex, living situation, education, smoking, and alcohol consumption were collected with a standardized protocol during the patient interview. Data regarding cancer diagnosis, neoadjuvant treatment, nutritional status, comorbidities, ASA-classification, surgical approach, perioperative data, and tumor stage (based on pathoanatomical diagnosis) were retrieved from the medical record. Three questions, recommended by the Swedish National Board of Health and Welfare, were used to screen for malnutrition.^35^ Comorbidity burden was calculated using the age-standardized Charlson Comorbidity Index,^36^ where the primary diseases (ie, colorectal adenocarcinoma and colorectal liver metastases) were not included. To assess independence in activities of daily living (ADL), the ADL Staircase questionnaire was used.^37^

### Outcomes

Outcomes were measured at 3 timepoints: baseline, before surgery (after the intervention) and after surgery, at discharge. The primary outcome, meters walked during the 6MWT, estimates functional capacity, where the test person is instructed to walk as far as possible for 6 min, back and forth on a 30-m course.^38^ Secondary outcomes were maximal inspiratory pressure (MIP) and performance on the 30-s chair stand test (30-s CST). MIP was assessed with the respiratory pressure meter Micro RPM (Care Fusion, San Diego, California, USA), which provides an estimate of inspiratory muscle strength,^39^ measured in cmH_2_O. The 30-s CST estimates lower extremity strength by measuring the number of sit-to-stands completed in 30 s without using the arms.^40^

### Statistical Methods

A power calculation (α = 5%, power = 0.80) for the 6 MWT, with an assumed SD of 111.5 m based on a subsample (those with low maximal walking speed) from a previous study,^23^ determined that 160 participants were needed to detect a difference of 50 m. Statistical analyses were conducted in IBM SPSS statistics version 29 and in R version 4.3.2,^41^ with additional R packages.^42-44^ We used an intention-to-treat approach, and a significance level (alpha) was set to 5% in all analyzes.

To analyze the effect of group allocation (intervention or control) at 3 timepoints (baseline, preoperative, postoperative), on outcomes of the 6MWT and MIP, we fitted 2 linear mixed-effects models (LMMs). Each LMM contained fixed effects of group allocation, timepoint and an interaction effect between group allocation and timepoint, as well as random intercept of patient ID. Post hoc pairwise comparisons on these models using least-square means revealed the difference between the 2 groups (intervention and control) at the different timepoints, as well as changes over time within each group. The resulting *P*-values were corrected with the Bonferroni correction for 9 comparisons, minimizing the risk of type 1 error. Model assumptions for these LMMs (normality, homogeneity of variances, influential outliers) were visually evaluated.^43^

The outcome 30-s CST was analyzed with 2 non-parametric Friedman rank sum tests, after generalized linear mixed effects models (Poisson GLMM and Negative Bionomal GLMM) were found to not adhere to model assumptions. The first Friedman test assessed differences in 30-s CST performance between groups across timepoints, while the second assessed differences within each group across timepoints.

Physical activity variables based on activPAL data were analyzed for between-group differences using Mann-Whitney *U* test due to non-normally distributed data. Median achieved intensity and number of performed sessions are presented for both supervised and unsupervised sessions, as well as percentage loading of MIP for supervised sessions. Compliance was measured as the percentage of participants conducting at least 6 supervised sessions and the percentage of planned supervised sessions completed. For descriptive statistics, continuous variables are presented as mean and standard deviations, ordinal data and skewed continuous variables as median and interquartile range, and categorical and binary data as frequencies and proportions.

## Results

### Recruitment

The study was conducted between April 2021 and June 2023. The recruitment pace was slow, mainly due to short waiting times for surgery and organizational factors, which resulted in difficulties performing the screening test. As a result, recruitment was discontinued in June 2023, due to financial reasons and time constraints.

### Participants

**Figure 1.**
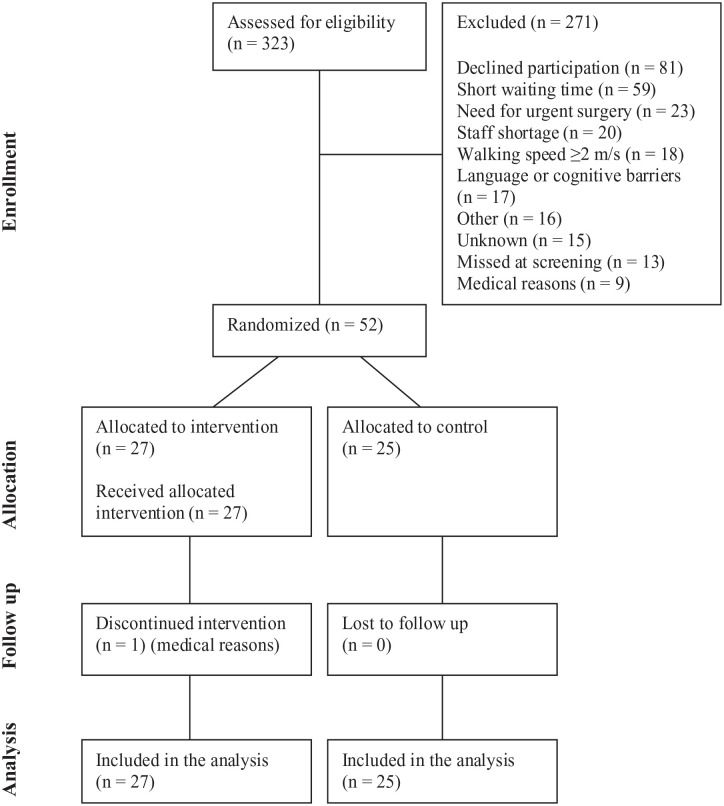
CONSORT flow diagram.

We assessed 323 patients for eligibility, of which 271 were excluded (Figure 1). Fifty-two patients were randomized, 27 to intervention and 25 to control group. All 52 participants were included in the analysis.

### Baseline Data

There were 28 (54%) female participants, and the mean ± SD age was 77 ± 6 years. The most common surgical approach for the whole sample was a minimally invasive procedure (61%; Table 1).

**Table 1.** Baseline Characteristics and Surgical and Oncological Data

Variable	Intervention (n = 27)	Control (n = 25)	Total sample (n = 52)
Demographics			
Age (years), mean (SD)	78 (4)	77 (7)	77 (6)
Female	16 (59)	12 (48)	28 (54)
Co-habiting^a^	13 (48)	16 (67)	29 (56)
University education^a^	15 (56)	7 (29)	22 (42)
Nutrition			
Risk of malnutrition^b^	8 (30)	11 (44)	19 (37)
Tobacco			
Never smoker	10 (37)	10 (40)	20 (38)
Alcohol			
No alcohol (or <1 standard glass/week)	15 (56)	10 (40)	25 (48)
Activities of daily living (ADL)			
Independence in ADL	15 (55)	12 (48)	27 (52)
Charlson Comorbidity Index			
2-3	9 (33)	12 (48)	21(40)
4-5	12 (44)	11 (44)	23 (44)
6-7	6 (22)	2 (8)	8 (15)
Cancer type, primary tumor			
Colon	19 (70)	18 (72)	37 (71)
Rectal	8 (30)	7 (28)	15 (29)
Neoadjuvant treatment			
Radiation	–	1 (4)	1 (2)
Chemotherapy	6 (22)	1 (4)	7 (13)
Chemoradiotherapy	2 (7)	3 (12)	5 (10)
ASA-classification			
I	1 (4)	1 (4)	2 (4)
II	9 (33)	14 (56)	23 (44)
III	17 (63)	10 (40)	27 (52)
Surgical site^a^			
Colon	16 (59)	14 (58)	30 (59)
Rectum	8 (30)	7 (29)	15 (29)
Liver^c^	3 (11)	3 (13)	6 (12)
Surgical approach^a^			
Open	11 (41)	9 (37.5)	20 (39)
Minimally invasive	16 (59)	15 (62.5)	31 (61)
Perioperative data^a^			
Bleeding (mL), Median (IQR)	150 (50-300)	175 (31-438)	150 (50-300)
Length of surgery (min), Median (IQR)	265 (161-307)	252 (149-396)	260 (160-361)
Tumor stage^a,d^			
0	–	2 (8)	2 (4)
I	3 (11)	4 (17)	7 (14)
II	11 (41)	7 (29)	18 (35)
III	8 (30)	6 (25)	14 (27)
IV	5 (18)	5 (21)	10 (20)

Abbreviations: ASA, American Society of Anesthesiologists physical status classification system; IQR, interquartile range; n, number; SD, standard deviation.

Data is presented as the number of participants (% per group or for the whole sample) unless stated otherwise.

a One case missing in the control group.

b Fulfilment of at least one of the following criteria indicate that there is a risk of malnutrition: unintentional weight-loss, eating difficulties, and low body mass index (<20 kg/m^2^ for patients <70 years of age and <22 kg/m^2^ for patients ≥70 years of age).^35^

c Colorectal liver metastases, including “liver first” approach (n=2) and simultaneous resection (n = 4).

d If >1 tumor was surgically removed, the highest staging is presented.

### Primary and Secondary Outcomes

**Figure 2.**
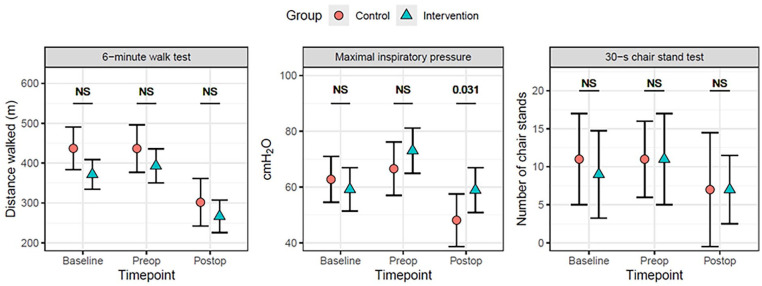
Shows the result for the primary and secondary outcomes. Abbreviations: Postop, postoperative; Preop, preoperative.

We found no significant differences between the intervention and the control groups for the primary outcome, the 6MWT (Table 2, Figure 2). Although the intervention group increased their 6MWT distance by 21 m from baseline to the preoperative assessment, this increase was not statistically significant (Table 2). Regarding secondary outcomes, a significant difference was found in MIP for the Group × Timepoint interaction effect (interaction effect, *F*_2, 81_ = 9.229, *P* < .001). Post hoc tests for MIP showed a significant difference between groups at the postoperative assessment (*P* = .031), favoring the intervention group. At baseline, the intervention group had lower values of 30-s CST, but for the pre- and postoperative assessment, values were similar between groups. However, there were no statistically significant differences in 30-s CST between or within groups (Table 2, Figure 2).

**Table 2.** Physical Fitness With Within and Between Group Comparisons Across Timepoints.

Variable	Intervention group, n = 27						Control group, n = 25						Between groups, *P*
	Baseline	Preop	P*	Postop	P**	P***	Baseline	Preop	P*	Postop	P**	p***	
	Mean (95% CI)^a^						Mean (95% CI)^a^						
6 MWT, m	372 (332-411)	393 (347-438)	.52	267 (222-311)	<.0001	<.001	437 (381-494)	437 (373-500)	1.00	302 (238-366)	<.001	<.001	.34^†^
	n = 26	n = 20		n = 16			n = 24	n = 21		n = 18			
MIP, cmH2O	59 (51-67)	73 (64-82)	<.001	59 (50-67)	1.00	<.001	63 (54-71)	67 (56-77)	1.00	48 (38-58)	<.001	<.001	.031
	n = 27	n = 22		n = 21			n = 25	n = 21		n = 19			
	Median (IQR)^b^						Median (IQR)^b^						
30-s CST, n	9 (7-13)	11 (8-15)		7 (3-9)			11 (7-15)	11 (9-14)		7 (2-11)			.08^‡^
	n = 26	n = 21		n = 19			n = 25	n = 21		n = 19			

Abbreviations: 6MWT, 6-min walk test; 30-s CST, 30-s chair stand test; CI, confidence interval; IQR, interquartile range; MIP, maximal inspiratory pressure; n, number.

a Linear mixed model.

b Friedmans test, no pairwise comparisons conducted.

* Baseline – preoperative.

** Baseline – postoperative.

*** Preoperative – postoperative

† Between group difference at the postoperative assessment.

‡ Between group difference across timepoints.

### Adherence

The median duration of the exercise intervention was 14 days (IQR = 13-16), and the median number of supervised sessions was 6 (range = 2-8). The proportion of participants conducting at least 6 supervised sessions was 67%, and 146 (90%) out of 162 planned supervised sessions were attended. One participant had a transient ischemic attack during the preoperative period, leading to a discontinued exercise intervention. Other reasons for fewer than 6 supervised sessions were: a late start of the exercise intervention (n = 3), national holidays during the intervention (n = 2), and illness or low energy (n = 3). For the supervised sessions, IMT achieved the desired median intensity of at least 5 on the Borg CR-10 Scale. Functional strength and aerobic exercise, however, had lower median intensity than aimed for (Table 3).

**Table 3.** Median Intensity Achieved and Median Number of Exercise Sessions During Supervised and Unsupervised Exercise.

Exercise type	Supervised exercise (n = 27)							Number of exercise sessions
	Total^a^	Session 1	Session 2	Session 3	Session 4	Session 5	Session 6	
	Intensity Borg CR-10, median (IQR)							Median (IQR)
IMT	5 (4-5.5)	4 (3-5)	4 (3-5)	5 (4-5)	5 (4-6)	5 (4-6)	5 (4-6)	6 (5-6)
Chair rise	5.5 (4.5-7)	4 (3-5.5)	5.5 (5-7)	6 (4.5-7)	6 (5-7)	6 (5-7)	6 (5-7)	5 (4-6)
Step-up	6 (5-7)	5 (4-6)	6.5 (5-7)	6.5 (5-7)	6 (5-7)	6 (4-7)	6 (4.5-7)	5 (4-6)
Aerobic	6.5 (5-7)	6 (5-7)	6 (5-7)	6 (5-7)	7 (6-7.5)	7 (5-7)	6 (5-8)	5 (4-6)
	% of Maximal Inspiratory Pressure, median (IQR)							
IMT	57 (50-64)	51 (50-57)	56 (50-70)	57 (55-66)	59 (52-74)	59 (56-73)	60 (56-84)	
	Unsupervised exercise (n = 27)							
	Intensity Borg CR-10, median (IQR) total^a^							
IMT	5 (4-6)							17 (13-22)
Chair rise	5 (4-6.5)							4 (1-7)
Step-up	7 (5.5-8)							1 (0-5)
Aerobic, Borg CR-10 < 5	3.5 (3-4)							3 (1-5)
Aerobic, Borg CR-10 ≥ 5	6 (5-7)							4 (2-8)

Abbreviations: IMT, inspiratory muscle training; IQR, interquartile range; n, number.

a Includes up to 8 supervised sessions. Two participants had more than 6 sessions, that is, 7 and 8.

Fifteen participants were prescribed weight-belts for chair rise exercises and 13 participants for step-up exercises during supervised sessions. The median weight was 3 kg (IQR = 2-4) for both chair rise and step-up. The median IMT load achieved during the supervised sessions was 57% (IQR = 50%-64%) of maximal capacity. The most common activities identified with the PSFS were stair-climbing (n = 9), walking longer distances (n = 5), and walking uphill/downhill (n = 5).

Regarding free-living physical activity in the preoperative phase, absolute values showed that the intervention group had a larger number of waking hours (*P* < .001) and time in sedentary behavior (*P* = .030). When analyzing relative values, the intervention group had a higher level of high-intensity physical activity (stepping time >100 steps/min) compared to the control group (*P* = .015; Table 4).

**Table 4.** Daily, Absolute, and Relative (Percentage in Relation to Waking Hours or Total Stepping Time) activPAL Values and Comparisons Between Groups.

Activity type	Intervention group, n = 19	Control group, n = 19	Between groups
Absolute values	Median (IQR)	Median (IQR)	*P*-value^e^
Steps, n	6374 (4268-7292)	4677 (2969-10 002)	NS
Sit to stand, n	47 (39-55)	44 (40-59)	NS
Standing, min	172 (146-272)	190 (127-209)	NS
Stepping time, min	78 (62-95)	62 (41-119)	NS
Stepping time 75-100 steps/min, min	39 (23-53)	30 (15-74)	NS
Stepping time >100 steps/min, min	9 (6-26)	3 (0-16)	NS
Sedentary behavior, min^a^	689 (633-797)	649 (548-676)	*P* = .030
Waking hours, min^b^	979 (955-1034)	920 (853-952)	*P* = .001
Relative values			
% Standing^c^	18 (14-24)	22 (15-22)	NS
% Stepping time^c^	7 (6-10)	7 (4-12)	NS
% Sedentary behavior^c^	73 (65-77)	72 (67-74)	NS
% Stepping time 75-100 steps/min^d^	50 (45-58)	47 (30-59)	NS
% Stepping time >100 steps/min^d^	14 (8-36)	2 (1-25)	*P* = .015

Abbreviations: IQR, interquartile range; min, minutes; n, number; NS, non-significant.

a Sitting, seated transport, and secondary lying.

b Standing, total stepping time, and sedentary behavior.

c Percentage of waking hours.

d Percentage of stepping time.

e Mann-Whitney *U* test.

### Adverse Events

One participant discontinued the exercise intervention after having a transient ischemic attack between exercise sessions, for which carotid artery stenosis surgery was indicated. Other AEs included a fall during a walk occurring between supervised sessions (not resulting in physical injury or discontinuation of the intervention) and transient dizziness reported in 4 sessions (2 events of light dizziness during IMT and 2 during step-up exercises). Musculoskeletal AEs reported were calf-pain (n = 1), knee-pain (n = 1), foot pain (n = 1), and increases in pre-existing hip-pain associated with increased walking distances (n = 1). For the participants experiencing calf- and knee-pain, pain was reported to decrease during the exercise period.

## Discussion

This study provides insight on the effect of a home-based, high-intensity preoperative exercise program on changes in physical fitness during the perioperative phase in older patients undergoing colorectal cancer surgery. Novel features are the inclusion of patients with low physical fitness based on screening of maximal gait speed,^23^ and the use of accelerometer data to report physical activity and sedentary behavior in both groups. Our findings showed no statistically significant impact of preoperative exercise on functional capacity or lower extremity strength but a significant effect on postoperative inspiratory muscle strength. Furthermore, at discharge, the intervention group had returned to baseline MIP values, whereas the control group had not.

Consistent with our results, Carli et al^45^ and McIsaac et al^46^ found no effect of preoperative exercise, as part of a multimodal approach, on pre- or postoperative performance in the 6MWT, among older frail patients undergoing colorectal cancer surgery. Per-protocol analyses in both studies revealed that the lack of effect may have been attributed to poor adherence. In our study, organizational factors limited the planned number of supervised sessions. Disregarding these factors, 85% of participants achieved the target of 6 supervised sessions. Frequent support from the physiotherapist and the ability to conduct exercise at home are elements of our intervention that may contribute to high adherence. In contrast to our study, Carli et al^45^ and McIsaac^46^ aimed for moderate-intensity exercise, which may be insufficient to generate meaningful adaptions in this short time frame.^47^ This also relates to our study, since we did not reach the target number of sessions and target intensities for aerobic and strength exercises. However, we did observe increases in the intervention group in the 6MWT and 30-s CST, which were not statistically significant. Despite the increases being relatively small, a 6MWT change of 20 m has previously been considered a clinically meaningful difference in this population.^48,49^ The short median duration of 14 days, together with lower exercise intensities than planned, may explain why increases in functional capacity and lower extremity strength were not greater.

A few studies have now been conducted on high-intensity exercise for high-risk patients undergoing major abdominal cancer surgery.^50-52^ These studies include 2 randomized controlled trials,^50,51^ and a prospective cohort study,^52^ all showing a positive impact on aerobic fitness or functional capacity.^50-52^ As these studies had longer program durations (≥3 weeks) compared to our study, it is reasonable to assume that their total exercise dose was higher. Studies demonstrating benefits of preoperative exercise, whether for high-risk patients or a more general population, use time frames incompatible with current treatment target times in Sweden. However, guidelines on treatment target times vary among countries with no clear consensus on the optimal time frame,^16^ and while short waiting times are often used to indicate care quality, more flexible guidelines may support structured interventions to improve the patients’ preoperative status.^53,54^ Baseline data on physical fitness in our study indicate that our screening tool was effective in identifying patients with low physical fitness. For this specific patient group, comorbidities or low physical capacity may pose challenges to reach standardized intensities, and a longer time frame may be needed to reach progression goals.

Although the intervention group had a higher median daily step count, there was no significant difference between groups. This was probably due to the wide range in the control group, suggesting that a few individuals were very physically active during the preoperative phase. Nevertheless, the intervention group had a median step count of over 6000 steps per day, which aligns with findings of a recent meta-analysis, suggesting a decreased risk of all-cause mortality with increasing daily steps, with a plateau around 6000 to 8000 steps per day for older adults.^55^ As a high-level of physical activity at the time of diagnosis may be associated with disease-free survival in patients with colorectal cancer, a potential goal of preoperative exercise could be to increase pre- and postoperative physical activity.^56^ Considering the nature of our exercise intervention, it is noteworthy that there was no significant difference in the daily number of sit-to-stand transfers between groups. Similar to our study, it is common that preoperative exercise is compared to enhanced usual care, typically with the addition of more general physical activity recommendations. While this can ensure standardized advice on physical activity between groups and sites, it may lead to alterations of the control groups behavior potentially mitigating the effect of a more structured exercise program. For example, Carli et al (2010) found that their control intervention, focusing on advice on walking and breathing exercises, was more effective than their structured exercise intervention in improving performance on the 6MWT.^57^ In addition to general physical activity recommendations, repeated measures of physical fitness and awareness of an ongoing accelerometer-based assessment of physical activity, may have led our control group to increase their level of physical activity. However, when analyzing relative values of high-intensity physical activity, there was a significant difference favoring the intervention group which may indicate that the intervention was effective in increasing this domain. While the activPAL may be particularly useful for this population, due to its ability to measure steps of slower walking speed or steps taken with a walking aid,^32^ one limitation is its tendency to underestimate time spent in higher intensity physical activity.^31^ Although this should be considered when interpreting adherence, it seems reasonable to assume that the intervention group engaged more in high-intensity physical activity.

It is not surprising that IMT led to a significant increase in inspiratory muscle strength from baseline to the preoperative assessment for the intervention group, as target intensity was achieved for self-perceived exertion and percentage of MIP training load. Furthermore, adherence appeared high for unsupervised sessions. Short-term IMT has previously been reported to increase preoperative inspiratory muscle strength in patients undergoing gastrointestinal surgery.^58^ Similar to more conventional strength training, IMT has been shown to improve inspiratory muscle strength by promoting hypertrophy of the targeted muscles.^59,60^ However, given the short time frame of our intervention, neural adaptions may account for the observed improvements.^61^ The potential clinical benefits of the observed increases in inspiratory muscle strength in our study remains unclear, however, IMT has previously been shown to lower the risk of postoperative pulmonary complications and reduce length of hospital stay in patients undergoing cardiac and major abdominal surgery.^62^

The main methodological weakness of our study is that we did not manage to recruit enough participants to achieve sufficient statistical power to detect a difference in our main outcome, and our results may therefore be subject to type II error. Despite our efforts to address participation barriers identified in the feasibility study, we encountered new challenges. Short waiting times for surgery, where postponement was not possible due to logistic reasons, significantly hindered recruitment. Additionally, the short waiting times influenced the median duration of the intervention. which was at the lower end of the planned duration. We also had a high decline-rate which increases the risk of selection-bias. Although our outcome assessors were blinded to group allocation for most of the study, we had to refrain from this at one instance when one of the trial group members conducted a preoperative assessment. Additionally, the sealing of the accelerometer may have hinted at group allocation, as accelerometers were removed and replaced for the intervention group. In this study, AE were rigorously reported, but one limitation is the lack of report on AE in the control group, which makes any interpretations regarding the safety of an intervention more tentative. However, studies on high-intensity preoperative exercise for high-risk patients have reported no or few, mainly minor, adverse events so far.^50,52^ Strengths of our study include the pragmatic approach with screening and preoperative exercises possible to implement into existing organizations. Furthermore, the accelerometer-based assessment of physical activity in both groups allows valuable comparisons.

## Conclusions

In this study focused on older patients with low physical fitness undergoing colorectal cancer surgery, we found a beneficial effect of preoperative exercise on postoperative inspiratory muscle strength, which was preceded by a significant increase in the preoperative phase. However, we were unable to demonstrate that preoperative exercise influenced pre- or postoperative functional capacity or lower-extremity strength. These results must be interpreted with caution due to the small sample size. Our results also suggest that preoperative exercise may be challenging to implement when organizational factors, rather than patient needs, determine the time frame available for preoperative optimization.
